# A new approach to recycle oxalic acid during lignocellulose pretreatment for xylose production

**DOI:** 10.1186/s13068-018-1325-3

**Published:** 2018-12-05

**Authors:** Banggui Cheng, Xiao Zhang, Qixuan Lin, Fengxue Xin, Runcang Sun, Xiaohui Wang, Junli Ren

**Affiliations:** 1 State Key Laboratory of Pulp and Paper Engineering, South China University of Technology, Guangzhou, 510640 China; 2 Biotechnology and Pharmaceutical Engineering, Nanjing University of Technology, Nanjing, 211800 China; 3 Centre for Lignocellulose Science and Engineering, and Liaoning Key Laboratory Pulp and Paper Engineering, Dalian Polytechnic University, Dalian, 116034 China

**Keywords:** Biomass, Pretreatment, Xylose, Oxalic acid, Recycling

## Abstract

**Background:**

Dilute oxalic acid pretreatment has drawn much attention because it could selectively hydrolyse the hemicellulose fraction during lignocellulose pretreatment. However, there are few studies focusing on the recovery of oxalic acid. Here, we reported a new approach to recycle oxalic acid used in pretreatment via ethanol extraction.

**Results:**

The highest xylose content in hydrolysate was 266.70 mg xylose per 1 g corncob (85.0% yield), which was achieved using 150 mmol/L oxalic acid under the optimized treatment condition (140 °C, 2.5 h). These pretreatment conditions were employed to the subsequent pretreatment using recycled oxalic acid. Oxalic acid in the hydrolysate could be recycled according to the following steps: (1) water was removed via evaporation and vacuum drying, (2) ethanol was used to extract oxalic acid in the remaining mixture, and (3) oxalic acid and ethanol were separated by reduced pressure evaporation. The total xylose yields could be stabilized by intermittent adding oxalic acid, and the yields were in range of 46.7–64.3% in this experiment.

**Conclusions:**

This sustainable approach of recycling and reuse of oxalic acid has a significant potential application for replacing traditional dilute mineral acid pretreatment of lignocellulose, which could contribute to reduce CO_2_ emissions and the cost of the pretreatment.

## Background

Biomass is regarded as a promising candidate to substitute for petroleum-based products such as fuels [1, 2], chemicals [3, 4] and polymer materials [5, 6] because of its abundance and renewability [7, 8]. Hemicellulose, as one of the main components in lignocellulosic biomass [9], can be hydrolysed into xylose [10]. Xylose is an important chemical which can be converted into the important platform chemicals and biofuels by chemical method or biotechnology [11–13]. Furthermore, xylose is a kind of non-caloric sweetener widely applied in food and beverage industries [14]. Therefore, the high-efficient production of xylose has become a hot topic in the area of biorefinery research.

Dilute acid pretreatment (DAP) was a conventional technology by which xylan-type hemicellulose in lignocellulose was hydrolysed to xylose-rich hydrolysate [11, 15, 16]. Generally, mineral acids such as H_2_SO_4_ [17], HCl [18] and H_3_PO_4_ [19] were used during traditional DAP. However, there are several disadvantages such as difficulty in acid recovery and serious corrosion of equipment [20]. Therefore, organic acids pretreatment had gradually drawn much attention [21–23]. Among plenty of organic acids, oxalic acid was found to have attractive catalytic performance for pretreatment because it could efficiently and selectively hydrolyse hemicellulose [20, 24–26]. Many researchers used oxalic acid to hydrolyse various feedstocks, such as corncob [20], bagasse [25], wheat straw [27], giant reed [28] and oil palm trunk [29]. Oxalic acid is a relatively inexpensive organic acid, but its price is still much higher than inorganic acid. However, oxalic acid was not recycled for reuse in most studies. Thus, the recovery of oxalic acid used in biomass hydrolysis is an urgent problem to be solved for reducing the cost of pretreatment.

The solubility of xylose was low in ethanol (298.2 K, 0.0076 ± 0.0001 g xylose/100 g ethanol) [30], but the solubility of oxalic acid was so high in ethanol (1 g oxalic acid could be dissolved in approximately 2.5 mL ethanol at room temperature). Therefore, xylose and oxalic acid in hydrolysate can be separated by ethanol extraction, which is depending on the different solubility. As an agricultural waste, corncob is rich in xylan-type hemicellulose, and widely is applied in food and biofuel industries. Herein, a new approach for the first time was brought forward to recycle oxalic acid during corncob pretreatment for xylose production. In this work, the dilute oxalic acid pretreatment (DOAP) of corncob was carried out, and oxalic acid used in the pretreatment could be recycled by ethanol extraction. The reuse of recycled oxalic acid during the corncob pretreatment was also investigated. In this research, oxalic acid used during the pretreatment could be recycled and reused, and ethanol used for extraction also could be recyclable, which contributed to the sustainable development of pretreatment and reduce the cost of the pretreatment. Moreover, in this work, used oxalic acid and ethanol could be derived from biomass [31, 32], which was beneficial to the closed loop recycle of carbon.

## Results and discussion

The reaction temperature, time and oxalic acid concentration had very important influences on the hydrolysis efficiency of corncob. Therefore, we firstly investigated the influences of reaction temperature, time and oxalic acid concentration on the hydrolysis efficiency and optimized the pretreatment conditions. Afterwards, the pretreatment with recycled oxalic acid (PROA) was conducted under the optimal pretreatment condition with the highest yield of xylose. Dewaxed corncob before pretreatment could obtain 396.69 mg glucose per 1 g corncob (mg/g), 313.64 mg/g xylose and 39.87 mg/g arabinose, which was determined according to the National Renewable Energy Laboratory (NREL) methodology [33].

### Influences of reaction temperature and time on hydrolysis efficiency

**Fig. 1:**
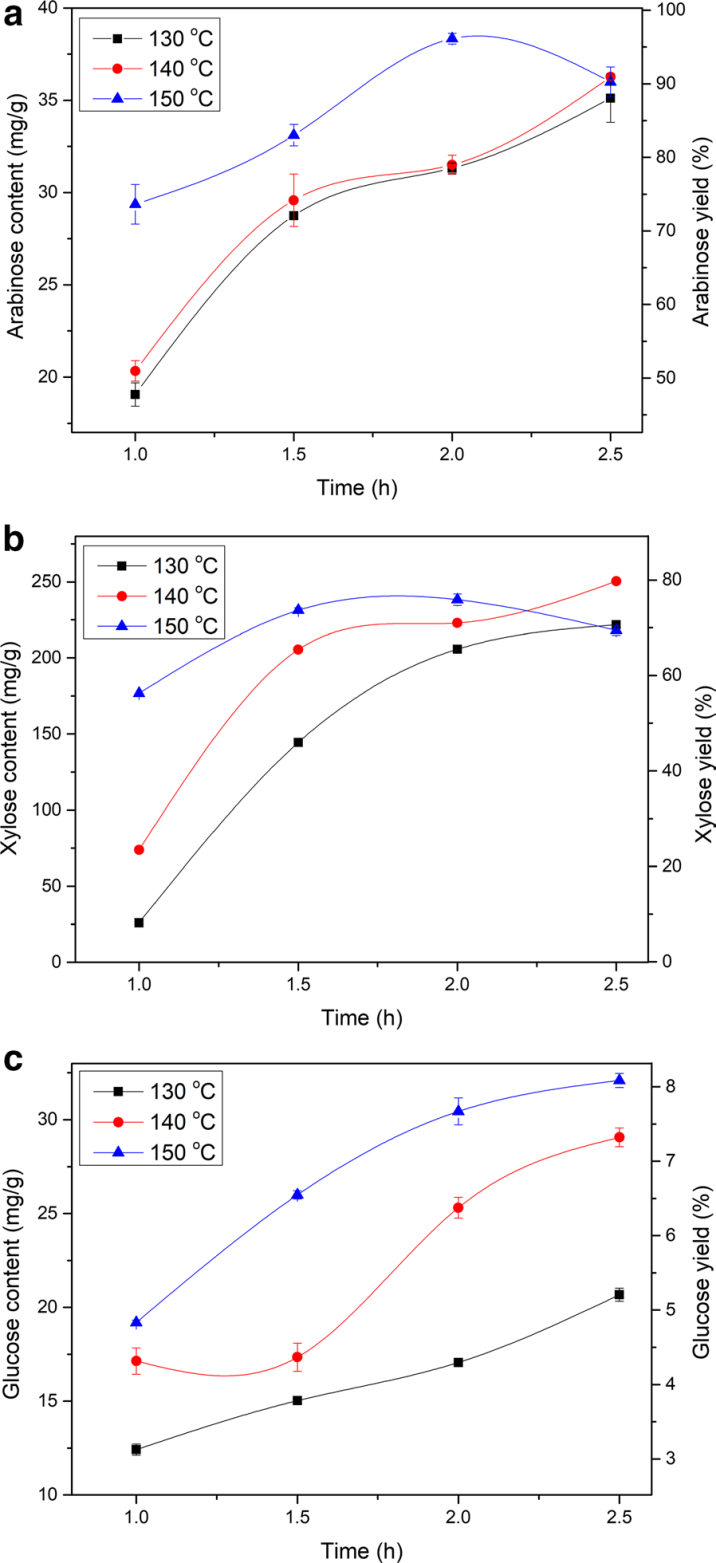
Effects of reaction temperature and time on sugar contents from hydrolysates. mg/g: mg per 1 g corncob. **a** Arabinose content, **b** xylose content and **c** glucose content. The oxalic acid concentration was 100 mmol/L

The reaction temperatures of DOAP were 130, 140 and 150 °C, the reaction time of DOAP was 1.0, 1.5, 2.0 and 2.5 h, and the oxalic acid concentration was 100 mmol/L. After this pretreatment, the contents of arabinose, xylose and glucose in the hydrolysate are shown in Fig. 1. The contents of arabinose and xylose showed an increasing tendency under 130 °C and 140 °C, but they displayed the tendency of increasing firstly and then decreasing under the 150 °C pretreatment condition (Fig. 1a, b). Among these pretreatments, the maximum xylose content was achieved up to 250.48 mg/g under the pretreatment condition with 140 °C reaction temperature and 2.5-h reaction time, correspondingly, the arabinose and glucose contents were 36.28 mg/g and 29.06 mg/g, respectively. In other words, 79.9% xylose and 91.0% arabinose could be released from the cell wall of corncob, indicating that oxalic acid had the high selectivity to hydrolyse xylan-type hemicellulose into C_5_ sugars (xylose and arabinose), and the branched chain mainly arabinose was easily broken down when xylan as the main chain was destroyed [20]. Under 130, 140 and 150 °C pretreatment conditions, the content of glucose presented an increasing tendency (Fig. 1c). The possible reason of this result was that a part of cellulose was hydrolysed to glucose when the severe pretreatment conditions were used [20]. To get high pentose yield, the optimized reaction temperature and time were set to 140 °C and 2.5 h, respectively, and the compositions of the hydrolysate were investigated under the pretreatment conditions of 10, 50, 100, 150 and 200 mmol/L oxalic acid concentration.

### Influence of oxalic acid concentration on hydrolysis efficiency

**Fig. 2:**
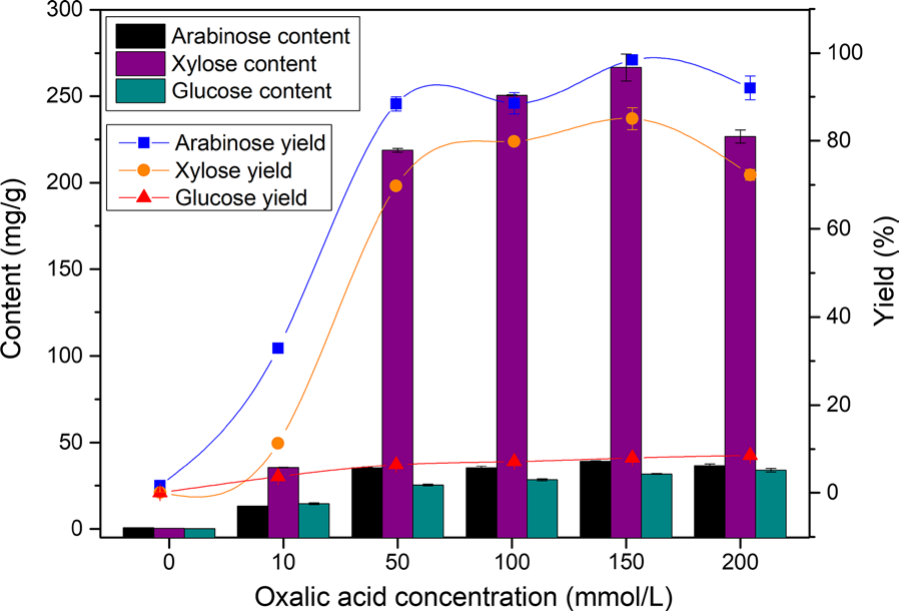
Effect of oxalic acid concentration on sugar contents. The reaction temperature and time were 140 °C and 2.5 h

After the pretreatments, the contents of arabinose, xylose and glucose in the hydrolysate were shown in Fig. 2. The contents of arabinose and xylose were increasing firstly and then decreasing, and the content of glucose was steadily increased. The hydrolysis efficiency was very low during pretreatment without addition of oxalic acid. As the concentration of oxalic acid increased, the contents of xylose and arabinose increased significantly. Among these pretreatments, the maximum content of xylose reached 266.70 mg/g, corresponding to 85.0% of yield under the conditions of 140 °C, 2.5 h and 150 mmol/L. Meanwhile, the arabinose content was achieved up to 39.25 mg/g, indicating that almost all arabinose without degradation was released into the hydrolysate. Correspondingly, the glucose content was 31.75 mg/g. These results confirmed that oxalic acid could selectively hydrolyse hemicelluloses during the corncob pretreatment. Deng et al. pretreated corncob with dilute oxalic acid to obtain 71.4% xylose yield, and this pretreatment required ball milling which was a process with high energy consumption. Barisik et al. optimized the reaction conditions of oxalic acid pretreatment and achieved a high yield (> 90%) under 210 °C reaction temperature and 3.6% (~ 400 mmol/L) oxalic acid concentration. In contrast, lower temperature and acid concentration were applied in this work, and relatively high yield of xylose (85.0%) could be obtained at 140 °C for 2.5 h using 150 mmol/L oxalic acid.

### Analysis of untreated and residual corncob

**Fig. 3:**
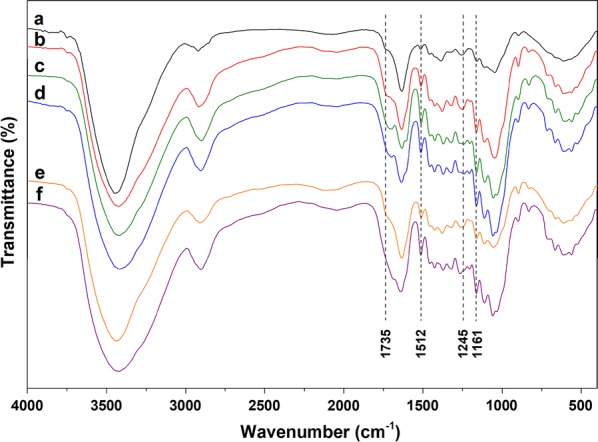
FTIR spectra of untreated and residual corncob. a: Untreated corncob, b pretreatment at 130 °C for 1.0 h using 100 mmol/L oxalic acid solution, c: pretreatment at 140 °C for 2.5 h using 100 mmol/L oxalic acid solution, d: pretreatment at 150 °C for 2.5 h using 100 mmol/L oxalic acid solution, e: pretreatment at 140 °C for 2.5 h using 10 mmol/L oxalic acid solution, f: pretreatment at 140 °C for 2.5 h using 200 mmol/L oxalic acid solution

After pretreatments, the residual corncob yields are shown in Table 1. Higher reaction temperature, longer reaction time and higher oxalic acid concentration could lead to a decrease in the residual corncob yield. Among these pretreatment conditions, oxalic acid concentration had the most significant influence on the residual corncob yields. When the oxalic acid concentration was 200 mmol/L, the corncob yield reached a minimum of 42.45%. These results revealed that a part of cellulose might be hydrolysed. The Fourier transform infrared spectra (FTIR) of untreated and residual corncob are displayed in Fig. 3. The bands around 1735 and 1245 cm^−1^ were attributed to stretch of C=O and C–O bonds for acetyl group in hemicellulose [4]. After the pretreatment, the intensities of the two bands in residual corncob were declined, indicating that hemicelluloses were hydrolysed to different extent. The band around 1161 cm^−1^ was attributed to vibration of C–O–C bond in cellulose and hemicellulose [34]. The band at 1512 cm^−1^ was ascribed to aromatic skeletal vibration in lignin [20], and the band in spectra of residual corncob was enhanced relative to the untreated corncob. The possible reason of this result was that the relative content of lignin and cellulose increased as hemicellulose was greatly removed from the cell wall of corncob.

**Table 1:** Effects of reaction temperature, reaction time and oxalic acid concentration on residual corncob yield

Reaction temperature (°C)	Reaction time (h)	Oxalic acid concentration (mmol/L)	Residual corncob yield (%)
130	1.0	100	64.76
130	1.5	100	54.66
130	2.0	100	50.31
130	2.5	100	50.06
140	1.0	100	56.98
140	1.5	100	52.16
140	2.0	100	49.27
140	2.5	100	47.15
150	1.0	100	53.47
150	1.5	100	49.4
150	2.0	100	47.03
150	2.5	100	44.72
140	2.5	0	77.64
140	2.5	10	55.93
140	2.5	50	50.18
140	2.5	100	47.09
140	2.5	150	46.14
140	2.5	200	42.45

**Fig. 4:**
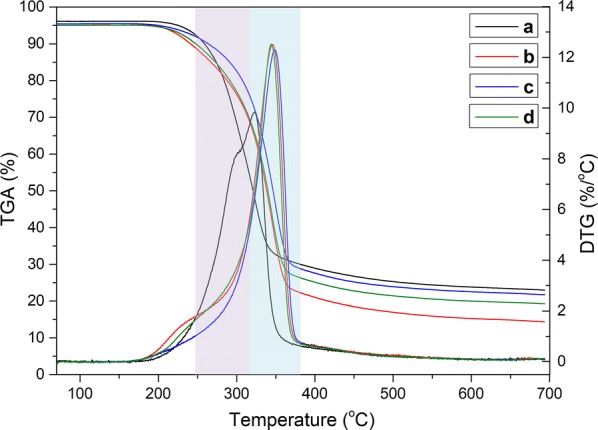
TGA and DTG curves of untreated and residual corncob. a: Untreated corncob, b: pretreatment at 140 °C for 2.5 h using 10 mmol/L oxalic acid solution, c: pretreatment at 140 °C for 2.5 h using 100 mmol/L oxalic acid solution, d: pretreatment at 130 °C for 1.0 h using 100 mmol/L oxalic acid solution

The curves of thermal gravity analysis (TGA) and differential thermogravimetry analysis (DTG) are shown in Fig. 4. In the curves of TGA, obviously, after the pretreatment, the thermal stability of corncob was increased because of the release of hemicelluloses from corncob. The main pyrolysis temperature range of hemicellulose, cellulose and lignin was 200–290 °C, 240–400 °C, 300–500 °C, respectively [25, 35]. The DTG curve of untreated corncob (Fig. 4a) had a shoulder peak at around 280 °C, and shoulder peaks in curves of residual corncob (Fig. 4b–d) became weaker or even disappear, which was attributed to the removal of hemicelluloses from the cell wall of corncob.

**Fig. 5:**
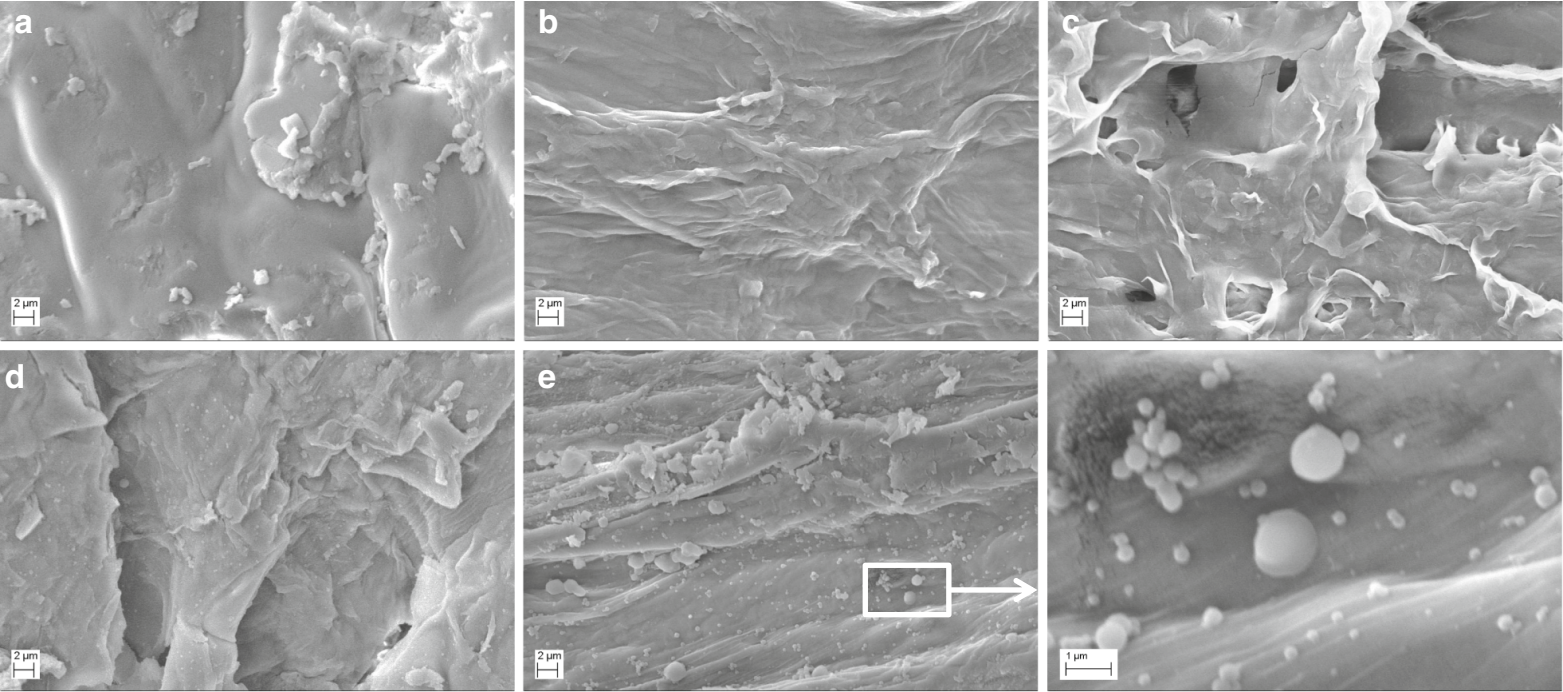
SEM images of untreated and residual corncob. **a** Untreated corncob, **b** pretreatment at 130 °C for 1.0 h using 100 mmol/L oxalic acid solution, **c** pretreatment at 140 °C for 2.5 h using 100 mmol/L oxalic acid solution, **d** pretreatment at 150 °C for 2.5 h using 100 mmol/L oxalic acid solution, **e** pretreatment at 140 °C for 2.5 h using 200 mmol/L oxalic acid solution

The scanning electron microscope (SEM) images are shown in Fig. 5. The surface of untreated corncob was smooth (Fig. 5a). After pretreatments, the surfaces of residual corncob were destroyed and a few cracks appeared (Fig. 5b–e), revealing that the original compact structure of lignocellulose was destroyed. When the pretreatment conditions were severe, microspheres would appear on the surface of the residual corncob (Fig. 5e). These microspheres might be lignin microspheres or pseudo-lignin microspheres which were formed from carbohydrates [36].

### Pretreatment with recycled oxalic acid

**Fig. 6:**
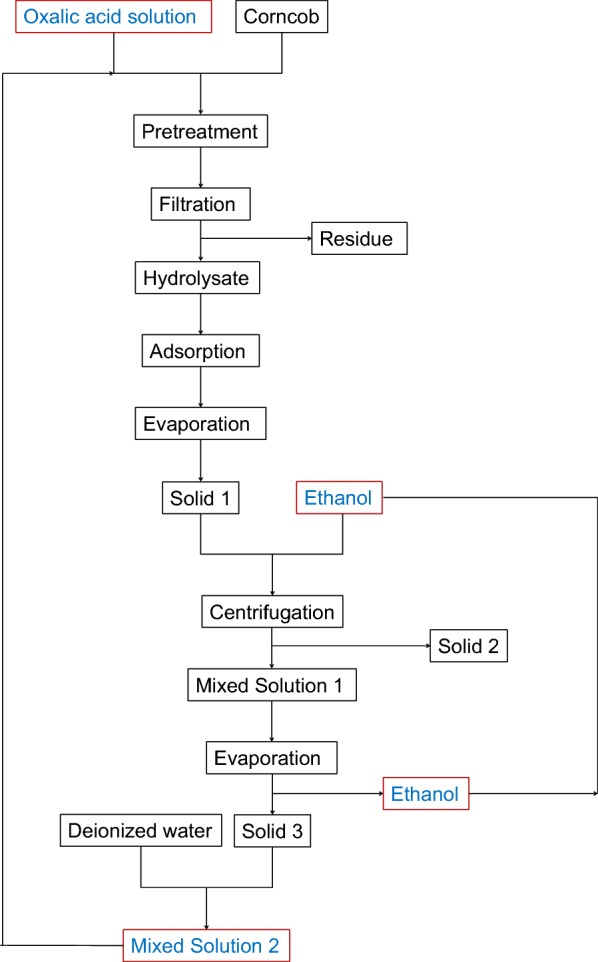
Flow diagram of pretreatment with recycled oxalic acid

**Fig. 7:**
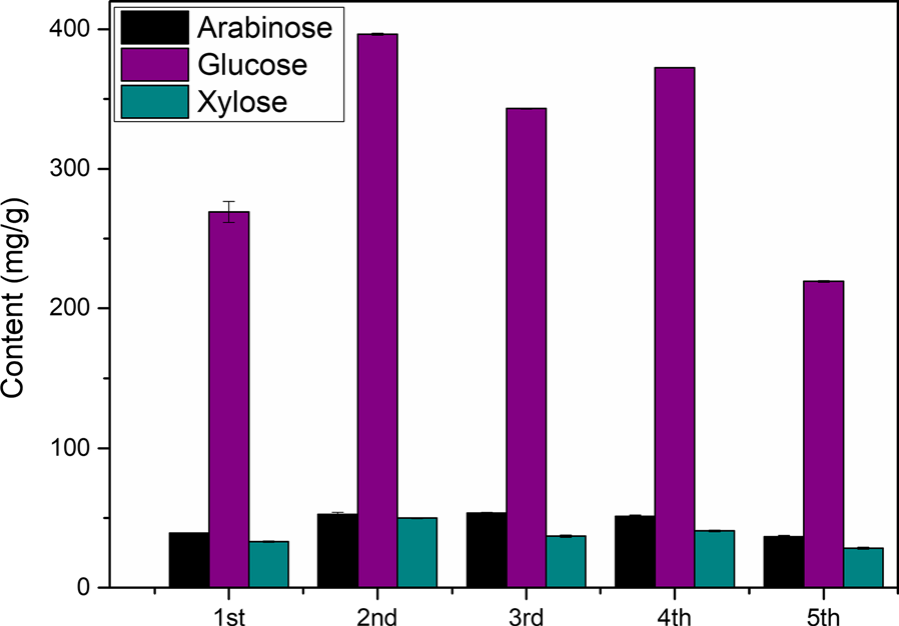
Sugar contents in hydrolysate for pretreatment with recycled oxalic acid. The reaction temperature and time were 140 °C and 2.5 h. 25 mL 150 mmol/L oxalic acid solution was additionally added at the preparation of Mixed Solution 2 for fourth cycle

**Fig. 8:**
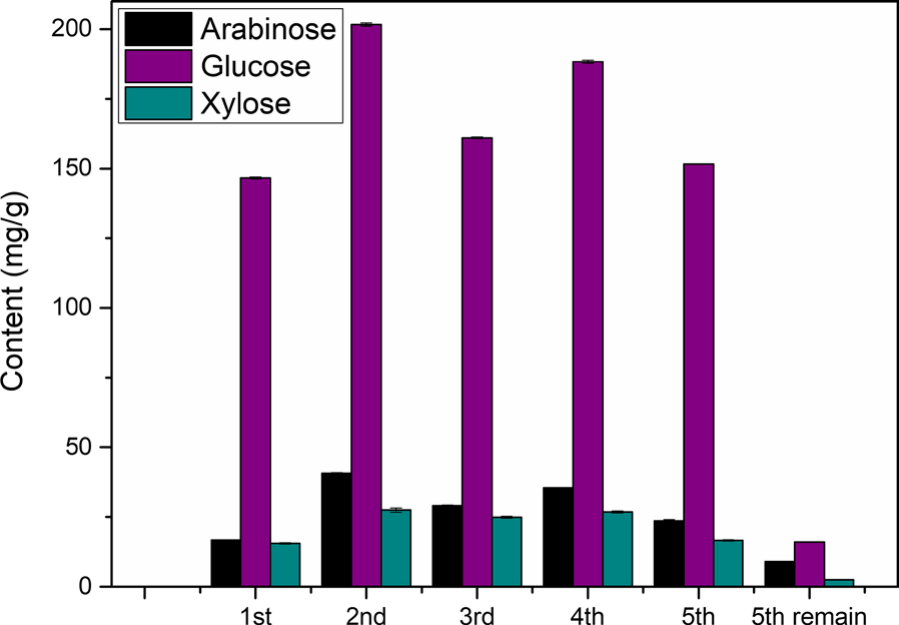
Sugar contents in Solid 2 for pretreatment with recycled oxalic acid. The reaction temperature and time were 140 °C and 2.5 h. 25 mL 0.150 mmol/L oxalic acid solution was additionally added at the preparation of Mixed Solution 2 for fourth cycle. The 5th remain was the residual Solid 3 after the fifth cycle

**Fig. 9:**
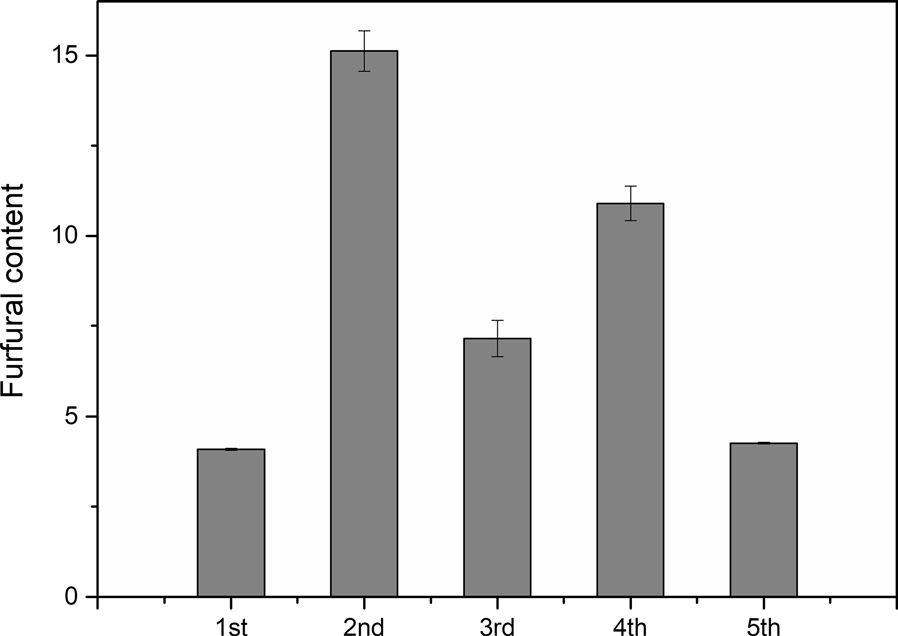
Furfural contents in hydrolysate for pretreatment with recycled oxalic acid

The conditions including reaction temperature of 140 °C, reaction time of 2.5 h and oxalic acid concentration of 150 mmol/L were employed for PROA. The flow diagram of PROA is displayed in Fig. 6. In this section, 1 g corncob and 20 mL oxalic acid solution were used for the pretreatment, and four groups were simultaneously conducted in parallel. Then, the hydrolysates were mixed and then used to subsequent procedure. To remove phenolic compounds from degradation of lignin, activated carbon was used to adsorb the phenolic compounds [37, 38]. Solid 1 obtained after the evaporation mainly contained xylose and oxalic acid. The evaporation step included evaporation at normal pressure and drying in a vacuum oven. The temperature of the vacuum drying was very important for the cycle process. High temperature of drying would cause side reactions such as the degradation of xylose, which would lead to a decrease in the total xylose yield. In this experiment, the temperature of the vacuum drying was set up to 30 °C. By the difference in solubility of xylose and oxalic acid in ethanol, ethanol was added to extract oxalic acid in Solid 1. After the centrifugation, Solid 2 and Mixed Solution 1 were obtained. Solid 2 mainly contained xylose, and the main components of Mixed Solution 1 were ethanol and oxalic acid. Solid 3 mainly containing oxalic acid was obtained via reduced pressure evaporation of Mixed Solution 1, and the ethanol used in extraction was recovered, simultaneously. Solid 3 was dissolved in 80-mL deionized water to prepare Mixed Solution 3 which was used for next pretreatment. The pretreatments were cycled for five times, and 25 mL 150 mmol/L oxalic acid solution was additionally added at the preparation of Mixed Solution 2 for fourth cycle. For PROA, the sugar contents in hydrolysate and Solid 2 are shown in Figs. 7 and 8, and the furfural content in hydrolysate is displayed in Fig. 9.

The tendencies were basically the same for the contents of xylose in hydrolysate and in Solid 2 (Figs. 7 and 8). During the evaporation step of hydrolysate, oxalic acid would first form oxalic acid dihydrate and then dehydrate to form anhydrous oxalic acid. In this evaporation step, the temperature was low (30 °C), and the oxalic acid dihydrate could not be completely dehydrated into anhydrous oxalic acid. Therefore, there a part of oxalic acid dihydrate in Solid 1. After the oxalic acid dihydrate was dissolved in ethanol, the crystal water in the oxalic acid dihydrate was mutually soluble with ethanol to form a solvent portion of the Mixed Solution 1. Compared with the first pretreatment, it increased obviously for xylose contents of the second pretreatment in hydrolysate and Solid 2. The reason of the result was that water contained in oxalic acid dihydrate for Solid 1 and ethanol would dissolve some xylose which entered into next pretreatment. Most of the dissolved xylose was still in the hydrolysate of next pretreatment, and a part of the dissolved xylose would be converted into furfural, as shown in Fig. 9 [39–41]. There were losses of oxalic acid during PROA, such as a part of oxalic acid entered into Solid 2. These losses led to a gradual decrease in the concentration of oxalic acid, resulting in a decrease in the efficiency of hydrolysis. Therefore, the xylose contents of the third pretreatment were less than the second. Compared with the third pretreatment, the xylose contents of fourth pretreatment increased because the additional addition of oxalic acid made the oxalic acid concentration increased. For the fifth pretreatment, the decreases of xylose contents were due to the losses of oxalic acid. Furthermore, byproduct such as humins from previous cycle would lead to decreases in the xylose contents [36]. After the fifth cycle, there are still some xylose, arabinose and glucose in residual Solid 3 (Fig. 8), which verified that a part of xylose, arabinose and glucose would be dissolved into water contained in oxalic acid dihydrate for Solid 1 and ethanol.

The contents of xylose in Solid 2 for the five cycles pretreatment were 146.55, 201.65, 160.98, 188.32 and 151.63 mg/g, and the corresponding total xylose yields were 46.7, 64.3, 51.3, 60.0 and 48.4%, respectively. Meanwhile, the contents of arabinose for the five cycles pretreatment were 16.74, 40.68, 29.07, 35.47 and 23.62 mg/g, and the contents of glucose were 15.64, 27.44, 24.90, 26.79 and 16.65 mg/g. The contents of total sugar, including xylose, arabinose and glucose, were 178.93, 269.76, 214.96, 250.58 and 191.90 mg/g. These results indicated that the yields could be stabilized via intermittent adding oxalic acid. Comparing dilute mineral acid pretreatment, corrosion of equipment was mild for DOAR. Oxalic acid and ethanol were recycled during PROA and activated carbon could be regenerated by carbonization, which contributed to reduce the cost of the PROA.

In the cyclic pretreatment process, the total xylose yields need to be further improved by increasing the hydrolysis efficiency of the cyclic pretreatment. Some significant factors will be discussed in the future work such as the amount of ethanol added to Solid 1, the amount of oxalic acid recovered from each recycling process and the amount of the supplementary oxalic acid solution in the pretreatment of corncob.

## Conclusions

In this work, a sustainable approach for the first time was developed to recycle oxalic acid used during the corncob pretreatment, and the recycled oxalic acid subsequently could be reused for corncob pretreatment. For the dilute oxalic acid pretreatment of corncob, the optimal conditions were 140 °C reaction temperature, 2.5-h reaction time and 150 mmol/L oxalic acid concentration, correspondingly, the xylose content in hydrolysate was achieved to 266.70 mg/g (85.0% yield) under these conditions. Oxalic acid used in pretreatment could be recovered via ethanol extraction. The total xylose yields for the five cycles pretreatment were in the range of 46.7–62.3%, which indicated that the xylose yields could be stabilized by intermittent adding oxalic acid. Meanwhile, the total sugar contents were 178.93–269.76 mg/g. This proposed approach contributed to reduce CO_2_ emissions and the cost of the pretreatment, and was promising for replacing traditional dilute mineral acid pretreatment.

## Methods

### Materials

Corncob used in this experiment was harvested from a farm in Dezhou, Shandong Province, China. Corncob was ground to 40–60 mesh and then extracted using the acetone/ethanol solution (2:1, V/V). Acetone (AR) and sulfuric acid (AR) were purchased from Guangzhou Chemical Reagent Factory (Guangzhou, China). Oxalic acid dehydrate (AR), ethanol (AR) and activated carbon (adsorption value of iodine: 100–800 mg/g) were purchased from Tianjin DAMAO (Tianjin, China), Guangdong Guanghua (Guangzhou, China) and Kermel (Tianjin, China), respectively. All the chemical reagents were used without further purification.

### Oxalic acid pretreatment

The DOAP was conducted in a hydrothermal reactor with stainless steel shell and polytetrafluoroethylene lining (Qiangqiangyiqi, Shanghai, China). The reaction temperatures were 130, 140 and 150 °C, the reaction time were 1.0, 1.5, 2.0 and 2.5 h, and the oxalic acid concentrations were 10, 50, 100, 150 and 200 mmol/L. The solid–liquid ratios were 1:20 (g/mL) for all the pretreatments. Corncob and the oxalic acid solution were placed in the reactor and heated to setting temperature. After the reaction was completed, the reactor was cooled to room temperature via ice water. The residue and hydrolysate were separated by G4 filter with bore diameter 3–4 µm.

### Residual corncob yield, FTIR, TGA and SEM analysis

The residual corncob yield was calculated based on the following Eq. 1:

1 $${\text{Residual corncob yield}} = \frac{{m_{\text{residual corncob}} }}{{m_{\text{total corncob}} }},$$

where *m*_residual corncob_ and *m*_total corncob_ were the mass of residual corncob and total corncob (1 g). FTIR spectra were recorded on a TENSOR27 spectrometer (Bruker, Germany) at room temperature, and the weight ratio of the sample to KBr was constant at 1:100, approximately. The thermal stability of sample was determined using a thermogravimetric analysis (TA Q500, America). The temperature program of TGA was heating from 30 to 200 °C, cooling to 50 °C and heating to 700 °C. The heating and cooling rates were 10 °C/min. The TGA and DTG curves displayed the program of heating to 700 °C from 70 °C. The morphology of sample was observed by SEM (ZEISS LEO1530VP, Germany), and the voltages of SEM was 10 kV.

### Recovery of oxalic acid and ethanol

For recovery of oxalic acid and ethanol process, 4-g activated carbon was added to the hydrolysate for adsorption at 80 °C for 30 min, and the separation of activated carbon and hydrolysate was conducted by G4 filter. Afterwards, the hydrolysate was concentrated to approximately 10 mL by evaporation at normal pressure and then dried in a vacuum oven (YIHENG, Shanghai, China) at 30 °C for 24 h, obtaining Solid 1. The oxalic acid in Solid 1 was dissolved via adding 15 mL ethanol, and then Solid 2 and Mixed Solution 1 were separated by centrifuge (SIGMA, Germany) at 4000 rpm for 10 min. Mixed Solution 2 was evaporated under reduced pressure at 30 °C using rotary evaporator (BUCHI, Switzerland). The ethanol in Mixed Solution 2 was evaporated and then condensed into a collection bottle. After the evaporation, Solid 3 was obtained. Mixed Solution 2 (80 mL) was prepared by dissolving Solid 3 using deionized water. Fresh corncob and Mixed Solution 2 were used for the next pretreatment.

### Analysis of sugar contents in liquid

All the liquid samples were detected three times with high-performance liquid chromatography (HPLC) and then averaged. The separated hydrolysate was diluted to an appropriate concentration and filtrated by 0.22-μm syringe filter. The sugar contents in Solid 2 were determined according the following method: Solid 2 was dissolved into deionized water to form a solution, and then the solution was diluted to an appropriate concentration. The diluted liquid was detected using HPLC (Waters, America) with a Waters 1515 Pump and a Waters 2412 Refractive Index Detector. The chromatographic column was an aminex column HPX-87H (BIO-RAD). The mobile phase was 5 mM H_2_SO_4_, the running rate was 0.5 mL/min and the temperatures of detector and column were 40 °C and 60 °C, separately [25]. The arabinose and glucose yields in Figs. 1 and 2 were calculated based on the following Eqs. 2, 3:

2 $${\text{Arabinose yield in hydrolysate}} = \frac{{m_{\text{arabinose in hydrolysate}} }}{{m_{\text{arabinose obtained from raw corncob }} }} \times 100\% ,$$

3 $${\text{Glucose yield in hydrolysate}} = \frac{{m_{\text{glucose in hydrolysate}} }}{{m_{\text{glucose obtained from raw corncob }} }} \times 100\% ,$$

where *m*_arabinose in hydrolysate_ and *m*_arabinose obtained from raw corncob_ were the mass of arabinose in hydrolysate and obtained from corncob before pretreatment; *m*_glucose in hydrolysate_ and *m*_glucose obtained from raw corncob_ were the mass of glucose in hydrolysate and obtained from corncob before pretreatment. The xylose yield in hydrolysate and total xylose yield were calculated based on the following Eqs. 4, 5:

4 $${\text{Xylose yield in hydrolysate}} = \frac{{m_{{xylose{\text{ in hydrolysate}}}} }}{{m_{\text{xylose obtained from raw corncob }} }} \times 100\% ,$$

5 $${\text{Total xylose yield}} = \frac{{m_{{xylose{\text{ in Solid 2}}}} }}{{m_{\text{xylose obtained from raw corncob }} }} \times 100\% ,$$

where *m*_xylose in hydrolysate_, *m*_xylose obtained from raw corncob_ and *m*_xylose in Solid 2_ were the mass of xylose in hydrolysate, obtained from corncob before pretreatment and in Solid 2, respectively.

## Abbreviations

DAP: dilute acid pretreatment
DOAP: dilute oxalic acid pretreatment
mg/g: mg per 1 g corncob
NREL: National Renewable Energy Laboratory
PROA: pretreatment with recycled oxalic acid
